# Adsorptive-removal of bromothymol blue & keto-bromothymol blue from wastewater using antioxidant curcumin: thermodynamic assessment, kinetic and isotherm modeling

**DOI:** 10.1186/s13065-025-01444-y

**Published:** 2025-03-28

**Authors:** Samia M. Ibrahim, Ahmed F. Al-Hossainy, Asmaa Y. Wahman

**Affiliations:** Chemistry Department, Faculty of Science, New Valley University, El-Kharga, New Valley, 72511 Egypt

**Keywords:** Bromothymol blue dye, Keto-bromothymol blue dye, Curcumin, Adsorption, Kinetics

## Abstract

Our research objective is using a spectrophotometer method at a wavelength of 430 nm to explore the removal of bromthymol blue (BTB) & keto-bromothymol blue (KBTB) dyes utilizing curcumin (CUR) as an adsorbent. The impacts of several factors such as initial dye concentration, adsorbent dose, contact time, and temperature, were examined. The adsorption equilibrium data were assessed utilizing Langmuir and Freundlich, as well as an appropriate reaction mechanism, were put forth and discussed. CUR, (CUR -BTB) and (CUR -KBTB) dye were confirmed using Fourier Transform Infrared Spectroscopy (FTIR) and Scanning Electron Microscope (SEM) techniques. The highest percentages of curcumin elimination of BTB, KBTB were 43 & 90%, respectively, at 430 nm and 25 °C, and dye adsorption by the adsorbent increased with increasing initial dye concentration but decreased with increasing adsorbent dose. First-order kinetic models in elimination of BTB and KBTB with correlation 0.97 & 0.98, respectively, were fitted using the experimental data for removal of BTB & KBTB by CUR. This demonstrated that chemisorption, which involves valence forces through the sharing or exchange of electrons, is the rate-limiting phase. Hence, the exothermic nature of BTB adsorption onto CUR is indicated by the negative value of ΔH°(-54.216 kJmol^− 1^). Once more, the non-spontaneous nature of the adsorption process is indicated by the positive ΔG° value (+ 49.65 kJmol^− 1^). Furthermore, the non-affinity of CUR for BTB dye is illustrated by the -ve of change of entropy, ΔS° (-166.78 J/mol K).

## Introduction

Due to the rapidly expanding dye industry, a significant quantity of dye wastewater has been continually allow to leave the water in recent years [1], endangering both human health and the natural environment. Industrial effluent is mostly responsible for water contamination. Among the several synthetic dyes used in various applications are methyl orange, acriflavine, rhodamine B, and thymol blue [2].

Environmental protection requires keeping an eye on dye use in terms of legislation because many businesses employ chemical dyes to treat their final products [3]. The effluent from the dyeing company has to be sufficiently treated before being dumped into any body of water because it has a range of substances and coloring materials. However, because of their highly variable composition, dye house effluents are especially challenging to manage properly [4].

Dyes are added to pharmaceutical and nutritional supplement products for practical, psychological, and commercial reasons. Kids are the target market for a wide range of colored tablets, syrups, capsules (soft and hard gelatin), and multivitamin supplements precisely because of their existence. Additionally, the color of pharmaceuticals makes them easier to recognize at first glance. One common pH indicator for silk dyes is bromothymol blue (BTB) [5]. This molecule is a useful probe that can only be chemically broken down by free radical pathways and is not contaminated by direct oxidation. When it comes to smoother storage, easily soluble solids, and improved water and soil treatment efficiency-as demonstrated for some contaminants-it offers many more advantages over conventional oxidizing agents [6]. Using an alkaline medium with a pH of at least 12, potassium permanganate oxidation was used to manufacture the novel chelating agent KBTB quantitatively [7–10].

Oxidation reactions can be used to convert BTB into keto-bromothymol blue [7–10]. In addition to changing KBTB’s chemical structure, the ketone group may also change its optical and reactivity characteristics. Although BTB is frequently used as a pH indicator, based on its unique characteristics, KBTB may have specific uses. Physical, chemical, and biological treatment techniques are now widely used to eliminate dye from wastewater [11]. These techniques do, however, have problems, including high energy consumption, high expense, and a large number of hazardous byproducts. Because of the adsorption method’s easy operation, low cost, abundance of adsorbent materials, ease of recycling, and high efficiency, the majority of researches have focused on it [12]. Many adsorbents have been studied such as zinc curcumin oxide nanoparticles [13], curcumin formaldehyde resin [14] and used, including activated carbon, zeolite, orange peel, wheat shells, SiO_2_, metal–organic frameworks (MOFs), and so on [15]. Although activated carbon is a widely utilized adsorbent, its employment in the adsorption area is restricted by its high cost and non-renewable sources [16]. The drawback of using SiO_2_ and MOF adsorbents is that they can cause indirect environmental contamination while being prepared [17]. As a result, the search for substitute adsorbent is going quite well. Researchers have looked into a variety of substitute adsorbents, and biopolymers are one of them that are starting to show promise as a substitute for activated carbon. These biopolymers feature a large surface area, excellent mechanical stiffness, variable surface chemistry, porosity, and mild conditions regenerability. Chitosan, chitin, cellulose acetate, and other biopolymers are among the many that can be employed [18, 19]. Some nanoparticles were synthesized and be used as adsorbent, it the environmentally benign, cost-effective approach to the fabrication of valuable for removal of toxic dyes [20–24]. Overall, nanoparticles show promise for the removal of hazardous dyes from wastewater. They have a large adsorption capacity, quick adsorption kinetics, and may be regenerated and reused. Again, several compounds used as a good adsorbent for removal of poisonous dyes such as chemically treated date stones, Jujube shell, ZnO and TiO_2_, copper-impregnated fishbone hydroxyapatite catalyst and metal-organic framework (MOF)-derived magnetic nanocomposites [25–29]. Methodologies for the removal of Allura Red, a commonly used synthetic azo dye, from contaminated water sources include two primary technologies: adsorption and photocatalytic degradation [30].

Because it is used in foods, cosmetics, and medications, curcumin is highly significant. It is added to foods to add color, such as soups, fats, candies, meat items, and beverages. Additionally, it acts as an antioxidant to stop rancidity [31]. It is a medication used to treat stomach issues, lower cholesterol, kill bacteria, purify blood, and protect the liver. It has strong anti-inflammatory, anti-HIV, anti-cancer, anti-thrombosis, and anti-Alzheimer properties [32]. Its special bio-protective qualities support the skin’s ability to neutralize free radicals, delaying the ageing process and UV radiation damage [32]. The various functional groups found in curcumin, including parahydroxy, keto, and double bonds, are what give it its antioxidative, anti-inflammatory, anti-cancer, and anti-mutagen properties. It also helps to lessen the effects of COVID-19 [33], as well as its antiarthritic, antiatherosclerotic, antidepressant, and antiaging advantages [34]. Again, curcumin, a natural pigment derived from turmeric, has demonstrated potential as an adsorbent for the elimination of contaminants from water. It is easily obtainable from turmeric, rendering it a sustainable and cost-effective choice. Also, Curcumin’s molecular structure interacts with contaminants through many methods, making it effective against a variety of contaminants.

The purpose of this work is to investigate BTB and KBTB dyes the removal of hazardous BTB and KBTB dye from wastewater using curcumin as an adsorbent. The novelty of our work is using CUR in the removal of two toxic dyes which different in function group, -OH in case of BTB and C = O in case of KBTB. We observe removal efficiency of BTB & KBTB value of 43 & 90%, respectively. So, C = O group in KBTB increase the removal percentage. To confirm our results, we look at the quantity, initial dye concentration, adsorbents, contact time, temperature, and the use of the Langmuir adsorption isotherm model. In order to remove color from synthetic, usually numerous effluents, this research aims to further the search for inexpensive adsorbents and their potential.

## Experimental sections

### Devices

Cells with a 1 cm path length were utilized to measure absorbance utilizing a programme controller and an automated scanning Double-beam Perkin Elmer Lambda 750 UV-Vis spectrophotometer. To accelerate the phase separation, a centrifuge (FRONTIERTM 5000 SERIES MULTI, OHAUS) was employed.

## Materials

**Fig. 1:**
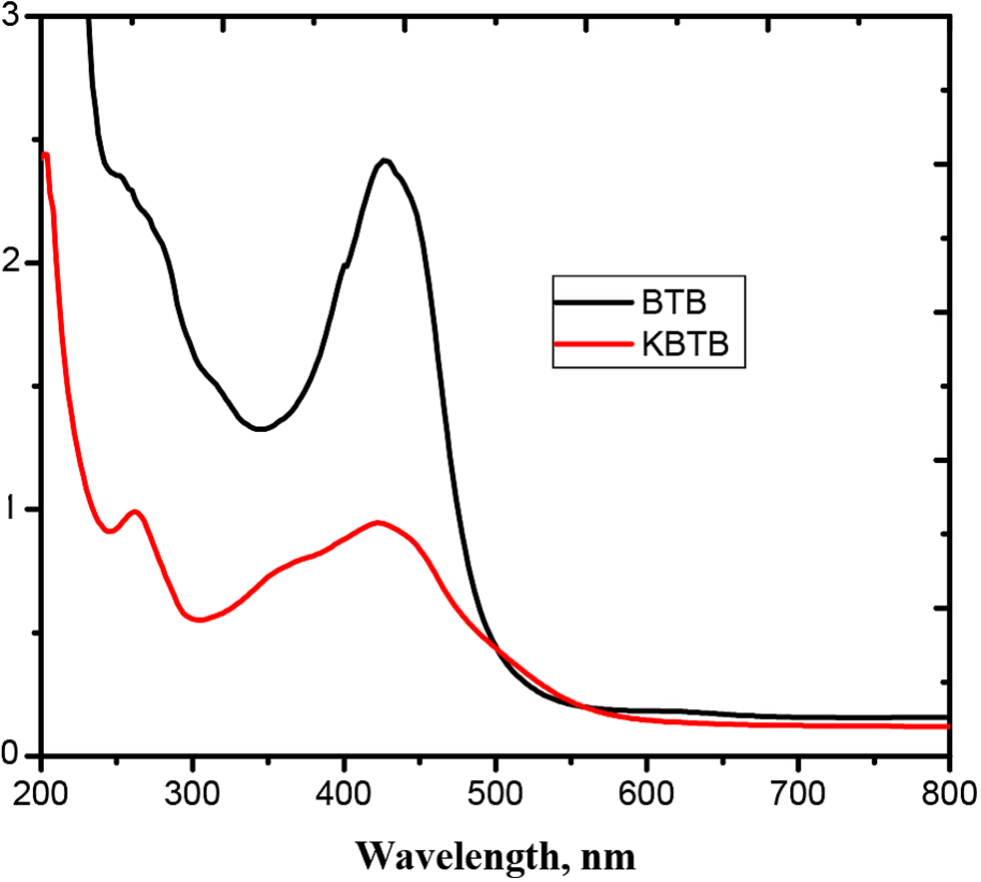
UV–Vis spectra of BTB & KBTB dyes

Throughout the entire study, analytical-grade materials were employed. Bromothymol blue and keto-bromthymol blue were used without further purification. To prepare liter of BTB & KBTB, precisely weighed volumes of dye were dissolved in 500 milliliters of distil water. The stock solutions were diluted in order to reach the necessary concentration. Every trial is carried out by brand-new dilutions. By diluting the stock solutions, the appropriate concentrations were obtained. Every experiment involved the use of fresh dilutions. By determining the dye’s concentrations, it was found at 430 nm wavelengths, as demonstrated in Fig. 1.

### Investigations of kinetic equilibrium and adsorption

The required strength of BTB & KBTB dyes stock solutions were achieved by diluting it. The curcumin adsorbent (0.1–1.2 gm) was examined at 25–55 °C, a contact time of 5.0–40 min, and a dye concentration of 30 mg L^− 1^. After every elimination condition test, the CUR, BTB and KBTB solutions were separated using centrifugation (2000 rpm, 10.0 min). The UV–Vis spectrophotometer was calibrated to measure the concentrations amount of remaining color molecules in the mixture at 430 nm. The kinetic examination included an assessment of the elimination efficiency. The capacity of adsorption (q_c_, mg g^− 1^) and removal efficiency (q_e_, %) were calculated using Eqs. (1) and (2), respectively [35, 36].

1 $$\:{\:\:q}_{c}=\:\frac{\left({C}_{0}-{C}_{e}\right)V}{m}$$

2 $$\:{q}_{e}=\:\frac{\left({C}_{0}-{C}_{e}\right)}{{C}_{0}}\:x\:100$$

Where C_0_ and C_e_ (mg L^− 1^), represents the initial and equilibrium liquid-phase dye concentrations, respectively. Where m is the mass of CUR utilized (g) and V is the volume of the BTB & KBTB solution (L).

### Adsorption technique

#### Langmuir model

The model of Langmuir’s can be applied to compute stable sorption in cases when the number of identical sites on the surface is finite. This is one possible format for it [37]:

3 $$\:\frac{1}{{q}_{c}}=\:\frac{1}{{q}_{m}}+\:\frac{1}{{K}_{L}{q}_{m}{C}_{e}}$$

The separation factor (RL) is another tool for characterizing this isotherm’s shape [38], which is calculated in the manner described below.

4 $$\:{\:\:\:\:\:\:\:\:\:\:\:\:\:\:\:\:\:\:\:\:\:\:\:\:\:\:\:\:\:\:\:R}_{L}=\:\frac{1}{1+\:{K}_{L}\:{C}_{0}}\:\:\:\:\:\:\:\:\:\:\:\:\:\:\:\:\:\:\:\:\:\:\:\:\:\:\:\:\:\:\:\:\:\:\:\:\:\:\:\:\:\:\:\:\:\:\:\:\:\:\:\:\:\:\:\:\:\:\:\:\:\:\:\:\:\:\:$$

A Langmuir parameter (L/mg) called K_L_ is associated with binding free energy and affinity of sorption. In this instance, the [BTB] & [KBTB] on the bio-sorbent at equilibrium (mg/g) is q_c_. C_e_ is a [BTB] & [KBTB] solutions at equilibrium (mg/l). The [BTB] & [KBTB] (mg/g) in a monolayer that forms on a bio-sorbent is denoted by q_m_.

#### Freundlich model

The equation of Freundlich method [38, 39] for systems with the heterogeneous of surface energy can be summarized as follows:

5 $$\:ln{q}_{c}=ln{K}_{f}+\:\frac{1}{n}\:ln{C}_{e}\:\:\:\:\:\:\:\:\:\:\:\:\:\:\:\:\:\:\:\:\:\:\:\:\:\:\:\:\:\:\:\:\:\:\:\:\:\:\:\:\:\:\:\:$$

ln q_c_ and ln C_e_ have a relationship that determines the Freundlich constants K_F_ and n. K_F_ and 1/n are the parameter constants that link the system’s sorption intensity and capacity. The term’s magnitude (1/n) represents the favorability of the sorbent/adsorbate systems [40–42].

## Results and discussion

### Adsorbent characterizations

#### FTIR analysis

**Fig. 2:**
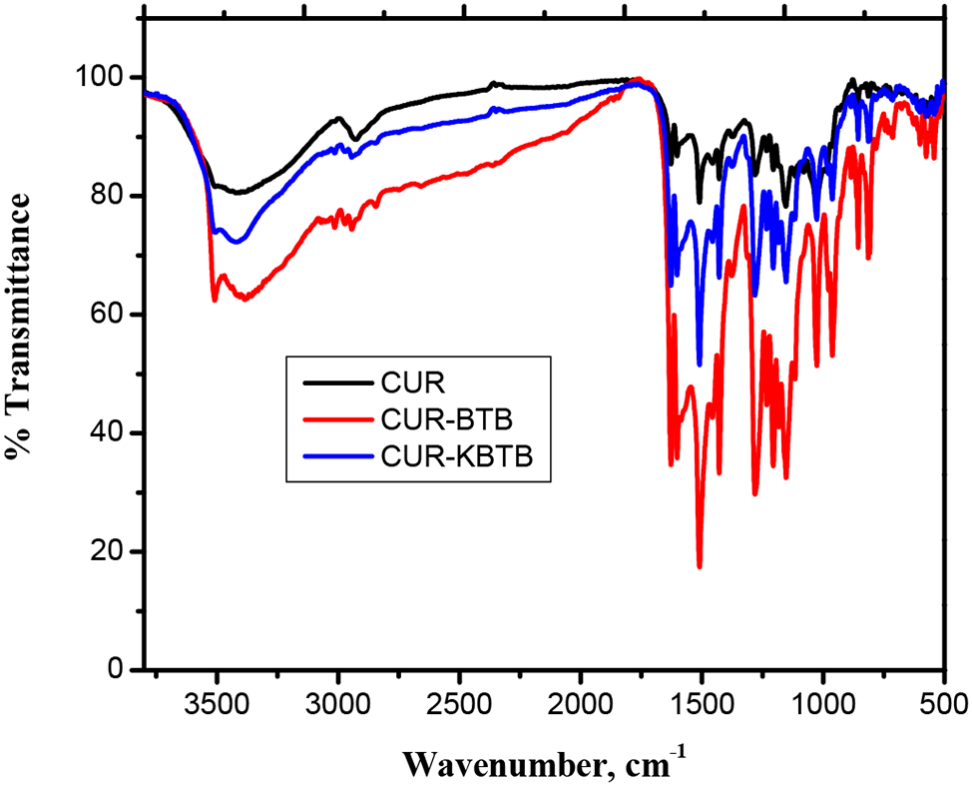
FTIR spectra of CUR, CUR-BTB and CUR-KBTB

The FTIR spectra of CUR and CUR-BTB & CUR-KBTB dyes (KBr), as displayed in Fig. 2, were highly diagnostic. The stretching frequency of the hydroxyl group, -OH, was thought to be responsible for the broad absorption band at approximately 3425 cm^-1^ prior to adsorption. The carbonyl group’s stretching vibration, represented by a moderately powerful band at 1624 cm^-1^, and the C-O stretching frequencies were represented by the other two bands, at 1285 and 1036 cm^-1^ [30]. While after adsorption, the FTIR spectra of CUR-BTB & CUR-KBTB changed significantly as shown in Fig. 2 [42, 43].

### SEM analysis

The morphological analysis has been performed with JSM5400 LV scanning electron microscopy. The morphological and textural surface properties of CUR are shown in Fig. 3 both before to and during BTB & KBTB adsorption. Therefore, the uneven structure of the surface and its numerous pores which may serve as adsorption sites in case of CUR are depicted in Fig. 3. It could also be helpful for the widespread distribution of dangerous dyes like BTB & KBTB. Significant layers of BTB & KBTB material was absorbed by CUR, resulting in the development of a BTB & KBTB materials coating on their surfaces (Fig. 3). The microstructures of CUR are altered by the physical and chemical interactions between the molecules of CUR and adsorbate. There have been other reports of similar incidents [43].

### CUR CUR- BTB CUR- KBTB

**Fig. 3:**
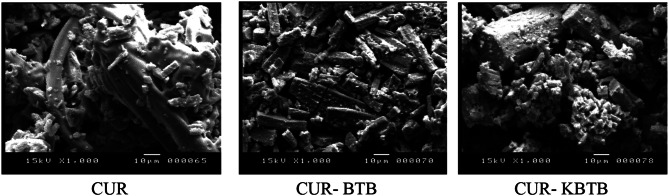
SEM micrographs of CUR: without adsorption, with BTB & KBTB adsorption

### Elimination of BTB at maximum wavelength equal to 430 nm

#### Impact of the starting [BTB] and CUR dosage

**Fig. 4:**
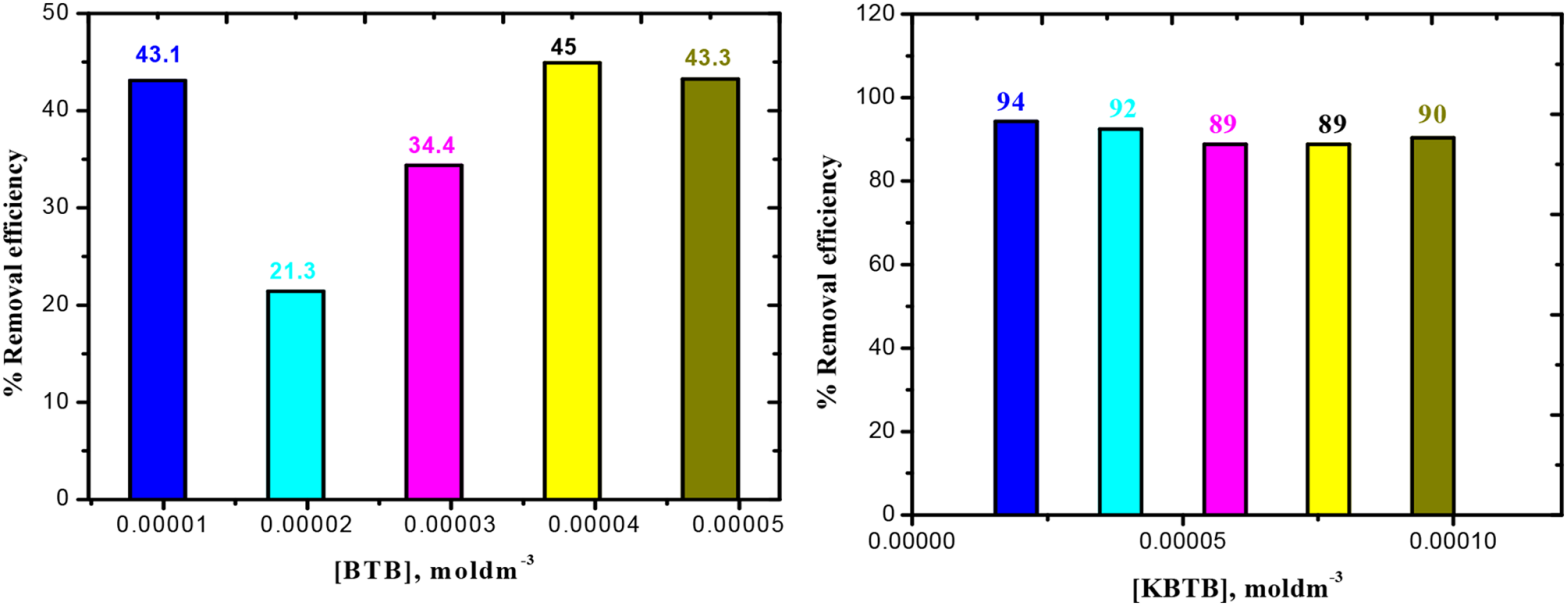
Effects of [BTB] & [KBTB] on removal efficiency in BTB removal at 25 ˚C

In range of (0.96–4.8) x10^− 5^ mol dm^− 3^, variations of [BTB]& [KBTB] were examined; the adsorption capacity equilibrium rose to a high value of 1.69 × 10^− 4^, mg/g. However, as [BTB] increase from 0.96 × 10^− 5^ to 4.8 × 10^− 5^ mol dm^− 3^, the % removal efficiency of BTB dye dramatically increased. Again, as [KBTB] increase from 1.92 × 10^− 5^ to 9.62 × 10^− 5^ mol dm^− 3^, the % removal efficiency of KBTB dye dramatically increased. It then decreased as BTB& KBTB dyes concentrations increased, reaching a maximum value of 45 & 94%, respectively, as illustrated in Fig. 4.

**Fig. 5:**
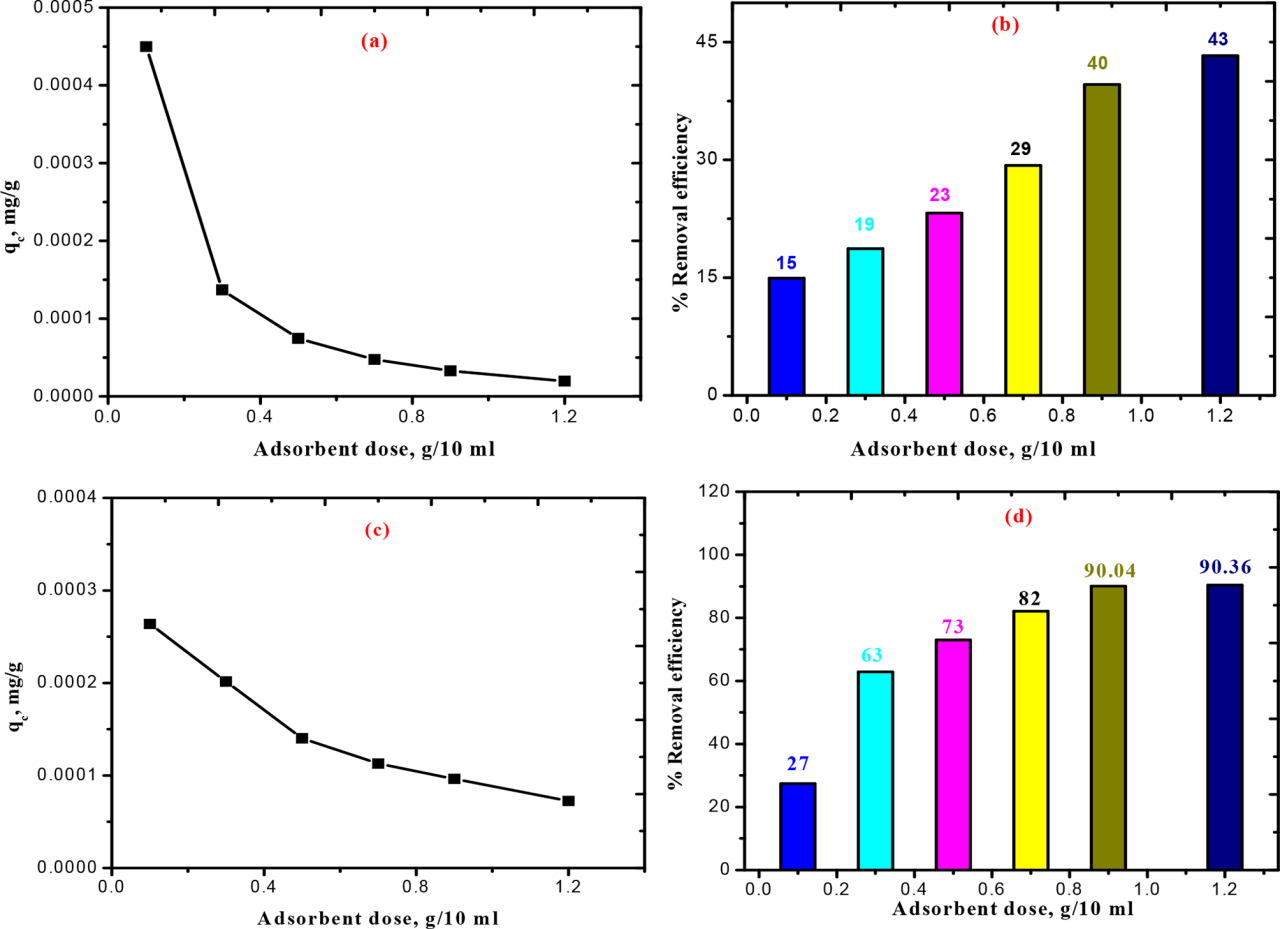
Dose of CUR effect on (**a**) capacity of adsorption (q_c_); (**b**) Removal efficiency on BTB; (**c**) capacity of adsorption (q_c_); (**d**) Removal efficiency on KBTB in 30 mg/l of BTB and 60 mg/l of KBTB at 25 ˚C

The adsorbents’ quantities varied from 0.1 to 1.2 g/10 ml. As the CUR amount is increased from 0.1 to 1.2 g/10 ml, Fig. 5 (a-d) illustrates that although the capacities of adsorption (q_c_) of the BTB & KBTB, respectively, rapidly reduce, the dye’s percentage removal efficiency of BTB & KBTB steadily increases to a maximum value of 43 & 90%, respectively.

### Variation of time

**Fig. 6:**
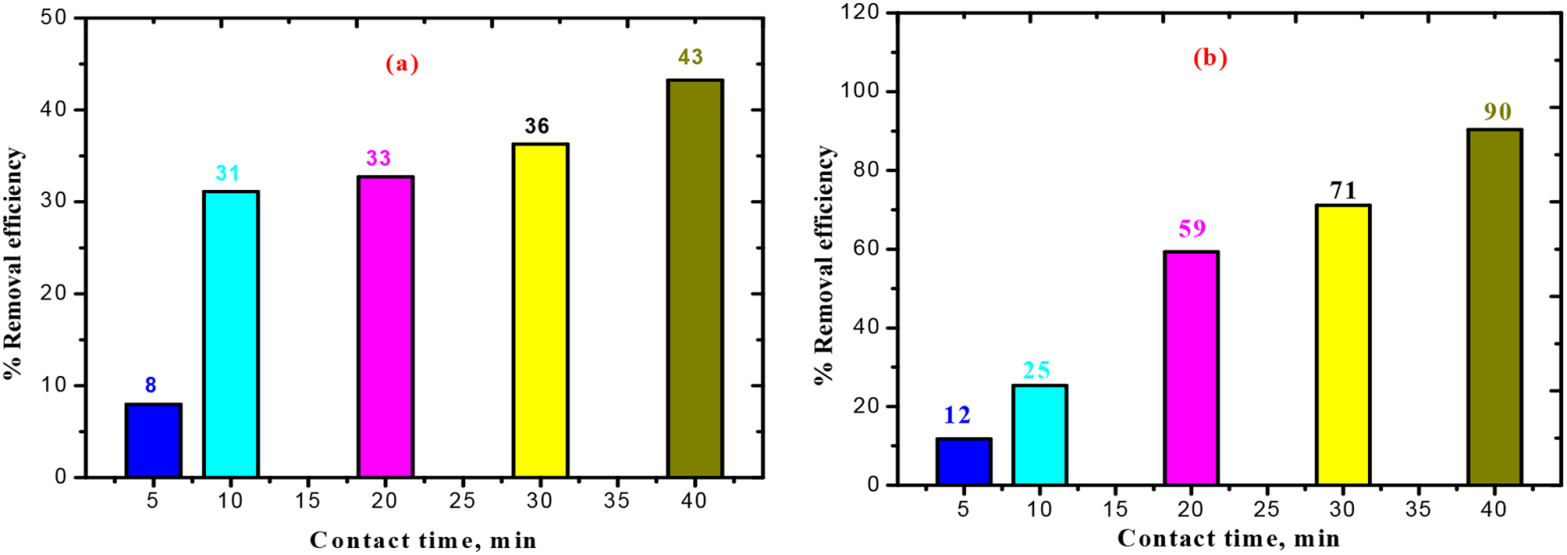
Time effect on efficiency of elimination in 30 & 60 mg/l of BTB & KBTB, respectively, at 25 ˚C

The impact of time on 30 & 60 mg/l of BTB & KBTB, respectively, elimination during a period of 5 to 40 min is depicted in Fig. 6 (a, b). Between 5 and 40 min, the capacity of adsorption and removal efficiency steadily increases to highest of 1.69 × 10^− 4^ mg/g & 43%, respectively in case of BTB and 7.24 × 10^− 4^ mg/g & 90%, respectively in case of KBTB. Once equilibrium was attained, these values stayed constant.

### Variation of temperature

**Fig. 7:**
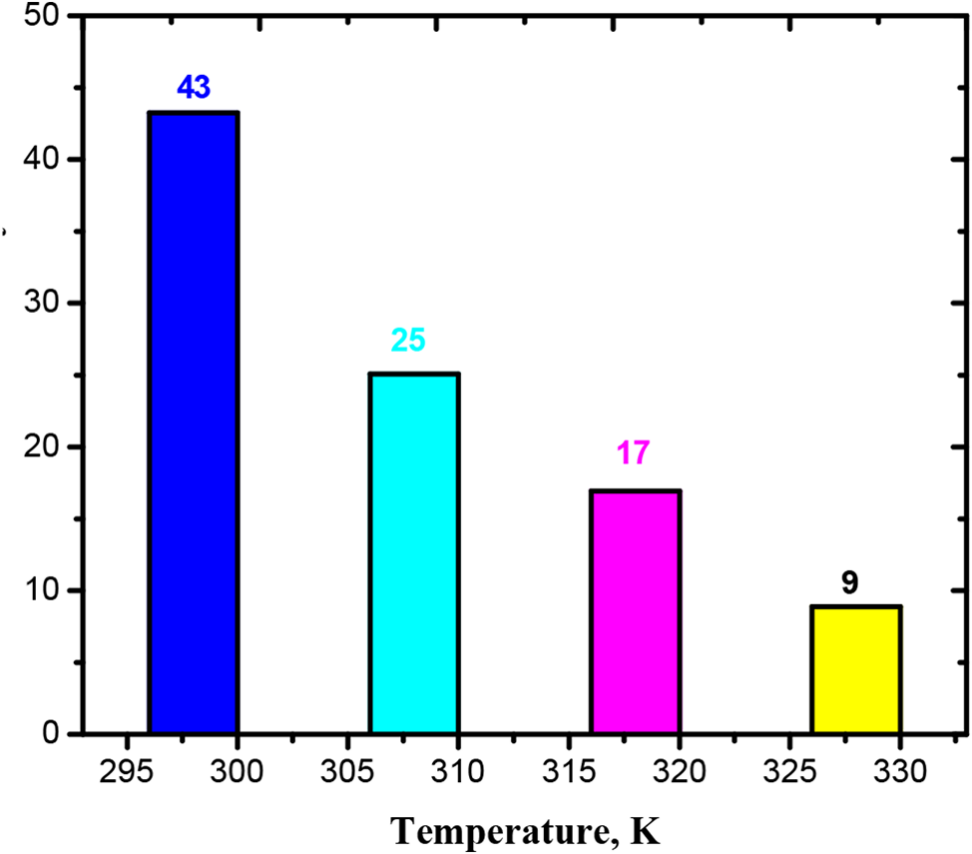
Variations of temperature on efficiency of removal in 30 mg/l of BTB

The dye’s adsorption capacity and removal efficiency decrease concurrently as the temperature increases from 25 to 55 °C, as illustrated in Fig. 7, with a maximum removal and adsorption capacity of 43% & 1.69 × 10^− 4^, respectively, at 298 K.

#### Isotherm of adsorption

The models such as Freundlich and Langmuir were employed to calculate the capacity of adsorption along with other constants. The capacity of sorption (q_m_) of elimination of BTB & KBTB were discovered to be 0.0003 & 0.0006 mg/g, respectively (Tables 1 and 2). The correlation coefficient of 0.98 confirms that the Langmuir isotherm, which assumes monolayer coverage and homogeneous activity distribution on the sorbent surface, can be utilized. Bromothymol blue & keto-bromthymol blue adsorption onto CUR are preferred in the present work by R_L_ data (0 < R_L_ < 1) (Tables 1 and 2). The Freundlich model was also fitted to the equilibrium data. The K_F_ and n constants, respectively, provided information on the system’s sorption intensity and capacity. The term (1/n) magnitude indicates the favorability of the sorbent/adsorbate systems [10, 40, 44–48].

**Table 1:** Data of several isotherm graphs for the elimination of BTB by CUR

Models	Isotherm constants			Correlation
Langmuir	**q**_**m**_ **(mg /g)**	**K**_**L**_ **(L/mg)**	**R**_**L**_	0.98
	0.0003	50315.23	0.3	
Freundlich	**n**	**K**_**F**_		0.99
	2.27	0.036		

**Table 2:** Data of several isotherm graphs for the elimination of KBTB by CUR

Models	Isotherm constants			Correlation
Langmuir	**q**_**m**_ **(mg /g)**	**K**_**L**_ **(L/mg)**	**R**_**L**_	0.99
	0.0006	454545.45	0.06	
Freundlich	**n**	**K**_**F**_		0.99
	2.66	0.039		

### Adsorption kinetics

The rate equation of 1st–order as:

6 $$\:\text{l}\text{n}\left({\text{q}}_{\text{c}}-{\text{q}}_{\text{t}}\right)=\text{l}\text{n}\:{\text{q}}_{\text{c}}-{\text{k}}_{1}\text{t}\:$$

where the first-order rate constant (min^-1^) is denoted by k_1_, and the dye quantities adsorbed at equilibrium and time t on the sorbent are represented by q_c_ and q_t_ (mg/g). By graphing ln(q_c_-q_t_) against t, the straight line can be derived.

The second-order rate expression’s linearized form is provided as [49]:

7 $$\:\frac{d{q}_{1}}{dt}=\:{k}_{2}{({q}_{e}-{q}_{e})}^{2}$$

8 $$\:\frac{t}{{q}_{t}}=\:\:\frac{1}{{k}_{2}{q}_{e}^{2}}+\:\frac{t}{{q}_{e}}$$

where k_2_ is the pseudo-second-order rate constant (g/mg min), q_e_ is the quantity of adsorbate adsorbed per unit mass of sorbent at equilibrium (mg/g), and q_t_ is the amount of adsorbate adsorbed at contact time t (mg/g). Plotting t/q_t_ against t not yields a linear connection [50].

First-order kinetic models in elimination of BTB and KBTB with correlation 0.97 & 0.98, respectively, were fitted using the experimental data for removal of BTB & KBTB by CUR, since this value has been identified for first-order kinetic models. This demonstrated that chemisorption, which involves valence forces through the sharing or exchange of electrons, is the rate-limiting phase [50–52].

The equation for intraparticle diffusion has the following expression:

9 $$\:{q}_{t}=\:{K}_{d}{t}^{1/2}+C$$

where K_d_, expressed in mg/g min^1/2^, is the intraparticle diffusion rate constant. The intraparticle diffusion experimental data of BTB & KBTB are 8.51 × 10^-6^ & 1.56 × 10^-4^ mg/g min^1/2^, respectively. The origin is not crossed by the plot’s linear section. This diversion from the intended course may result from variations in the mass transfer rate between the first and last phases of adsorption. The presence of outside surface adsorption and inner CUR diffusion indicates that the BTB adsorption process involved many steps [53].

### Adsorption thermodynamics

For BTB adsorption onto CUR, the calculated thermodynamic parameters are the enthalpy change (ΔH°), entropy change (ΔS°), and free energy change (ΔG°). This equation was used to calculate these parameters [54–63].

10 $$\Delta {{\rm{G}}^ \circ } = - 2.303{\rm{RT}}\log \;{{\rm{K}}_{\rm{D}}}$$

where K_D_ = q_c_/C_e_.

Also,

11 $$\Delta {{\rm{G}}^{^ \circ }} = \Delta {{\rm{H}}^{^ \circ }} - {\rm{T}}\Delta \;{{\rm{S}}^{^ \circ }}$$

12 $$\:\text{l}\text{n}{\text{K}}_{\text{D}}=\:\frac{\varDelta\:{\text{S}}^{^\circ\:}}{\text{R}}-\:\frac{\varDelta\:{\text{H}}^{^\circ\:}}{\text{R}\text{T}}$$

where R is the universal gas constant (8.314 J/mol K), C_e_ is the BTB concentration in solution at equilibrium, and q_e_ is the BTB concentration onto CUR (mg/L). Based on the slope and intercept of the plot of ln K_D_ against 1/T, the values of ΔH° and ΔS° were calculated. By Eq. (11) the change of Gibbs free energy (ΔG°) was estimated.

**Fig. 8:**
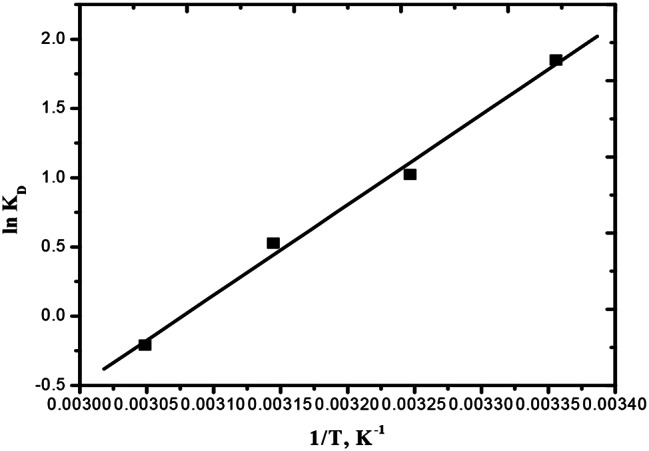
Plot of lnK_D_ against 1/T in the removal of BTB by CUR in 30 mg/l of BTB

Using Eqs. (10)–(12), the thermodynamic factors (ΔH°, ΔS°, and ΔG°) for elimination of BTB were determined. Based on the slope and intercept of ln K_D_ against 1/T (Fig. 8), the ΔH° and ΔS° values were calculated. Table 3 shows the thermodynamic constants for elimination of BTB by CUR. An exothermic reaction is indicated by the negative value of ΔH° (-54.216 kJmol^-1^). This is the result of BTB adsorption onto CUR. The estimated value of ΔG° (+ 49.65 kJmol^-1^) suggests that the adsorption method is non-spontaneous. Furthermore, the non-affinity of CUR for BTB dye is illustrated by the -ve of change of entropy, ΔS° (-166.78 J/mol K). The results obtained are summarized in Table 3.

**Table 3:** Thermodynamic constants for elimination of BTB at various temperatures

Parameter	*ΔH* ^*°*^ kJmol^− 1^	*ΔS* ^°^ Jmol^− 1^K^− 1^	*ΔG* ^°^ kJmol^− 1^
K_D_	-54.216	-166.78	+ 49.65

### The suggested mechanism of adsorption

**Fig. 9:**
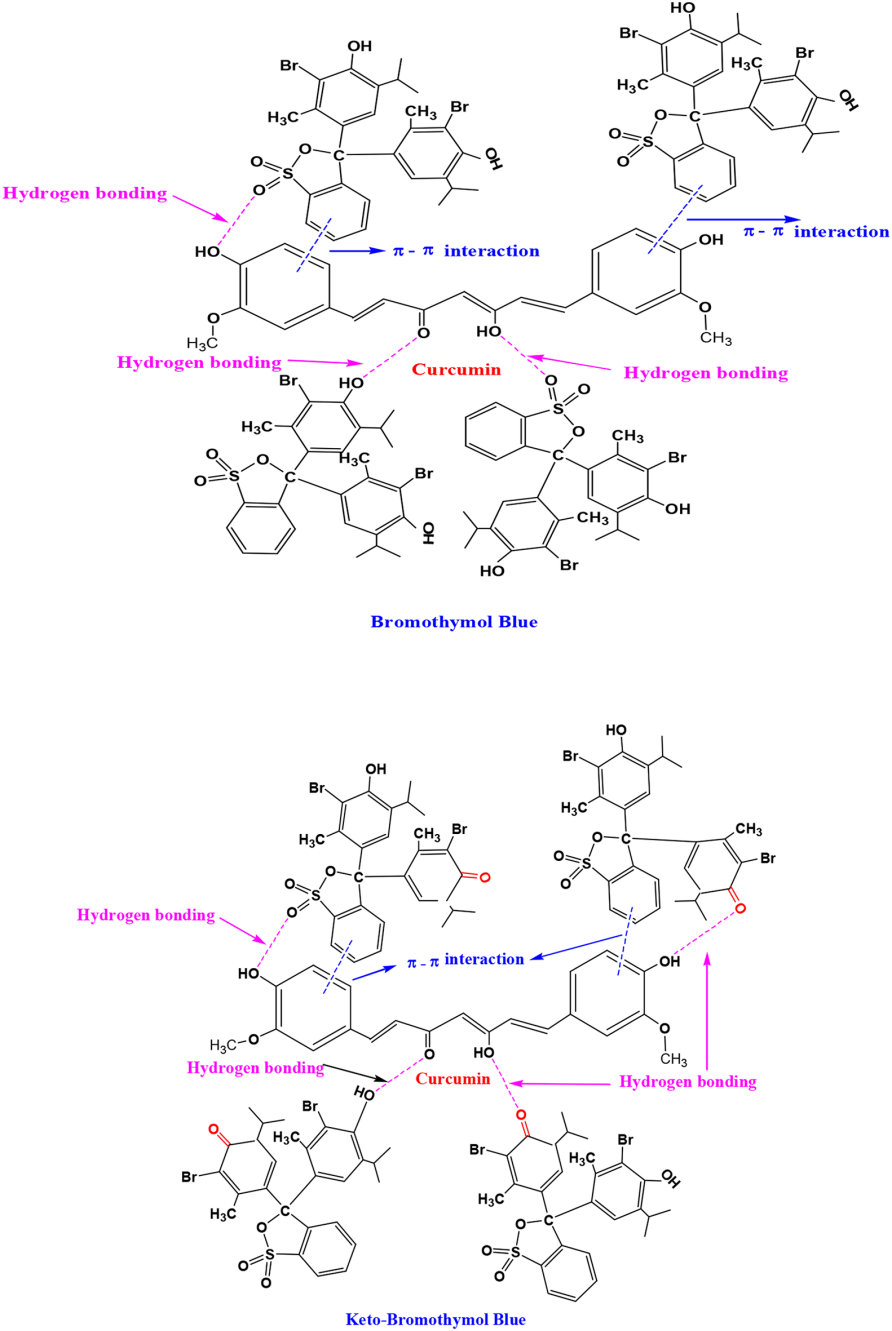
Speculated adsorption mechanism for the removal of BTB & KBTB by CUR

The chemical interactions between BTB & KBTB and the curcumin were suggested to be significant in the removal of BTB & KBTB based on the kinetics, models of adsorption, and thermodynamics of the adsorption results. Numerous interactions, such as hydrogen bonding, Van der Waals forces, electrostatic contact, and π-π interactions, are involved in the adsorption of cationic dyes [64]. Adsorption of BTB & KBTB onto CUR may occur π-π interactions and by hydrogen bonds formed between the hydroxyl group and the oxygen atom of the S = O group in the BTB & KBTB molecules and the hydroxyl group and oxygen atom of the carbonyl group on CUR. Because of this, the CUR may adsorb BTB & KBTB with a high capacity (Fig. 9).

## Conclusions

The present study shows the use of KBTB as adsorbent and compared with the BTB using CUR as considerably efficient for removal of dyes from wastewater. The FTIR spectra of CUR and CUR-BTB & CUR-KBTB dyes shows the stretching frequency of the hydroxyl group, -OH, was thought to be responsible for the broad absorption band at approximately 3425 cm^− 1^ prior to adsorption. The carbonyl group’s stretching vibration, represented by a moderately powerful band at 1624 cm^− 1^. While after adsorption, the FTIR spectra of CUR-BTB & CUR-KBTB changed significantly. Again, the morphological and textural surface properties of CUR shows the uneven structure of the surface and its numerous pores which may serve as adsorption sites in case of CUR. It could also be helpful for the widespread distribution of dangerous dyes like BTB & KBTB. Significant layers of BTB & KBTB material was absorbed by CUR, resulting in the development of a BTB & KBTB materials coating on their surfaces. The microstructures of CUR are altered by the physical and chemical interactions between the molecules of CUR and adsorbate. The adsorption is highly dependent on contact time, adsorbent dose and adsorbent concentration. The kinetics of BTB & KBTB dyes adsorption on CUR follows the First-order model. The equilibrium data fit well in the Langmuir and Freundlich models of adsorption, showing monolayer coverage of dye molecules at the outer surface of CUR. According to the acquired experimental data, the temperature increases from 20 to 50 °C, and as the temperature rises, so do the BTB adsorption capability and percentage removal. Once more, thermodynamic parameters were computed and examined.

## Data Availability

All data generated or analyzed during this study are included in this published article.

## References

[1] Pattnaik P, Dangayach. GS analysis of influencing factors on sustainability of textile wastewater: a structural equation approach. Water Air Soil Pollut. 2019;230:156.

[2] Chandhru M, Kutti Rani S, Vasimalai N. Reductive degradation of toxic six dyes in industrial wastewater using diaminobenzoic acid capped silver nanoparticles. J Environ Chem Eng. 2020;8:104225.

[3] Haddad E. Removal of basic Fuchsin dye from water using mussel shell biomass waste as an adsorbent: equilibrium, kinetics, and thermodynamics. J Taibah Univ Sci. 2016;10:664–74.

[4] Sivamani S, Grace BL. Removal of dyes from wastewater using Adsorption- A review. IJBST. 2009;2:47–51.

[5] Giasuddin ABM, Kanel SR, Choi H. Adsorption of humic acid onto nanoscale zerovalent iron and its effect on arsenic removal. Environ Sci Technol. 2007;41:2022–7. 17410800 10.1021/es0616534

[6] Xiang-Rong X, Hua-Bin L, Wen-Hua W, Ji-Dong. G decolorization of dyes and textile wastewater by potassium permanganate. Chemosphere. 2005;59:893–8. 15811419 10.1016/j.chemosphere.2004.11.013

[7] Al-Hossainy AF, Combined experimental. TDDFT-DFT computation, characterization, and optical properties for synthesis of keto-bromothymol blue dye thin film as optoelectronic devices. J Electron Mater. 2021;50:3800–13.

[8] Ibrahim SM, Al-Hossainy. AF kinetics and mechanism of oxidation of bromothymol blue by permanganate ion in acidic medium: application to textile industrial wastewater treatment. J Mol Liquids. 2020;318:114041.

[9] Al-Hossainy AA, Ibrahim A, Mogharbel RT, Ibrahim. SM synthesis of novel keto-bromothymol blue in different media using oxidation–reduction reactions: combined experimental and DFT-TDDFT computational studies. Chem Pap. 2021;75:3103–18.

[10] Al-Hossainy AF, Ibrahim. SM oxidation process and kinetics of bromothymol blue by alkaline permanganate. Int J Chem Kinet. 2021;53:675–84.

[11] de Oliveira GR, Fernandes NS, De Melo JV, Da Silva DR, Urgeghe C, Martínez-Huitle. CA electrocatalytic properties of Ti-supported Pt for decolorizing and removing dye from synthetic textile wastewaters. Chem Eng J. 2011;168:208–14.

[12] Zhang G, Shi L, Zhang Y, Wei D, Yan T, Wei Q, Du. B aerobic granular sludge-derived activated carbon: mineral acid modification and superior dye adsorption capacity. RSC Adv. 2015;5:25279–86.

[13] Arab C, El Kurdi R, Patra. D zinc Curcumin oxide nanoparticles for enhanced adsorption of congo red: kinetics and adsorption isotherms study. Mater Today Chem. 2022;23:100701.

[14] Alshehri SM, Naushad, Mu, Ahamad T, Alothman ZA, Aldalbahi. A synthesis, characterization of Curcumin based ecofriendly antimicrobial bio-adsorbent for the removal of phenol from aqueous medium. Chem Eng J. 2014;254:181–9.

[15] Hasanzadeh M, Simchi A, Far. HS nanoporous composites of activated carbon-metal organic frameworks for organic dye adsorption: synthesis, adsorption mechanism and kinetics studies. J Ind Eng Chem. 2020;81:405–14.

[16] Gusmão KAG, Gurgel LVA, Melo TMS, Gil. LF adsorption studies of methylene blue and Gentian Violet on sugarcane Bagasse modified with EDTA dianhydride (EDTAD) in aqueous solutions: kinetic and equilibrium aspects. J Environ Manage. 2013;118:135–43. 23428463 10.1016/j.jenvman.2013.01.017

[17] Zhu Z, Li G, Zeng G, Chen X, Hu D, Zhang Y, Sun. Y Fast capture of methyl-dyes over hierarchical amino-Co_0.3_Ni_0.7_Fe_2_O_4_@SiO_2_ nanofibrous membranes. J Mater Chem. A 2015; 3: 22000–22004.

[18] Hokkanen S, Bhatnagar A, Sillanpää. M A review on modification methods to cellulose-based adsorbents to improve adsorption capacity. Water Res. 2016;91:156–73. 26789698 10.1016/j.watres.2016.01.008

[19] Kanagaraj P, Nagendran A, Rana D, Matsuura. T separation of macromolecular proteins and removal of humic acid by cellulose acetate modified UF membranes. Int J Biol Macromol. 2016;89:81–8. 27118046 10.1016/j.ijbiomac.2016.04.054

[20] Ahmad W, Ahmed S, Kumar S. Facile one step microwave assisted biofabrication of Fe_2_O_3_ NPs: potential application as solar light-driven photocatalyst in the photodegradation of acridine orange. Int J Environ Anal Chem. 2024;104:25.

[21] Ahmad W, Joshi H, Ahmed S, Kumar S, Wilson I. Parmelia Perlata mediated microwave-assisted one-pot green synthesis of NiO nanoparticles a noble approach: antibacterial and photocatalytic activity evaluation. Chem Phys Lett. 2024;853:141524.

[22] Ahmad W, Ahmed S, Kumar S, Joshi. HC facile one step microwave-assisted bioextract-mediated green synthesis of ZnO NPs and subsequent investigation of their antibacterial and photocatalytic activity. Chem Pap. 2024;78:8309–20.

[23] Ahmad W, Joshi A, Kumar S, Rana R, Arora. A Bio-extract-mediated microwave-assisted synthesis of Cr_2_O_3_ nanoparticles: characterization, antibacterial, antioxidant, and photocatalytic activity evaluation. MRS Adv. 2024;9:970–8.

[24] Ahmad W, Kumar. Taxus Wallichiana leaf extract-mediated microwave-assisted one-pot biosynthesis of MgO NPs for biomedical and photocatalytic applications. Emergent Mater. 2024;7:1081–90.

[25] El Messaoudi N, El Khomri M, Bentahar S, Dbik A, Lacherai A, Bakiz. B evaluation of performance of chemically treated date stones: application for the removal of cationic dyes from aqueous solutions. J Taiwan Inst Chem Eng. 2016;67:244–53.

[26] El Messaoudi N, El Khomri M, Dbik A, Bentahar S, Lacherai A, Bakiz B. Biosorption of congo red in a fixed-bed column from aqueous solution using jujube shell: experimental and mathematical modeling. J Environ Chem Eng. 2016;4(Part A):3848–55.

[27] El Messaoudi N, Miyah Y, Imad Wan-Mohtar WAA, Hanafiah ZM, Ighalo JO, Emenike EC, Georgin J, Laabd M, Nouren L, Kausar A, Graba. B advancements in adsorption and photocatalytic degradation technologies of brilliant green from water: current status, challenges, and future prospects. Mater Toady Chem. 2024;42:102399.

[28] Miyah Y, Benjelloun M, Mejbar F, Ssouni S, El-Habacha M, Iaich S, El Messaoudi N, Zerrouq M, Souilah M, Lahrichi A, Zerrouq. F CWPO mechanism for toxic dye degradation in the presence of Cu@FbHAp catalyst: DFT study, performance analysis, response surface methodology, regeneration, and cost Estimation. Results Chem. 2025;13:102038.

[29] Miyah Y, El Messaoudi N, Benjelloun M, Georgin J, Stracke D, Franco P, Acikbas Y, Kusuma HS, Sillanpää. M MOF-derived magnetic nanocomposites as potential formulations for the efficient removal of organic pollutants from water via adsorption and advanced oxidation processes: A review. Mater Today Sustain. 2024;28:100985.

[30] El Messaoudi N, Miyah Y, Singh N, Gubernat S, Fatima R, Georgin J, El Mouden A, Saghir S, Knani S, Hwang. Y A critical review of allura red removal from water: advancements in adsorption and photocatalytic degradation technologies, and future perspectives. J Environ Chem Eng. 2024;12:114843.

[31] Stankovic I. Chemical and Technical Assessment. 61st Joint Expert Committee on Food Additives2004; 1: 1–8.

[32] Khanna N. Turmeric-nature’s precious gift. Curr Sci. 1999;76:1351–6.

[33] Babaei F, Nassiri-Asl M, Hosseinzadeh H. Curcumin (a constituent of Turmeric): New Treatment Option against COVID-19. Food Sci Nutr. 2020;8:5215–27. 33133525 10.1002/fsn3.1858 PMC7590269

[34] Aggarwal BB, Gupta SC, Sung B, Curcumin. An orally bioavailable blocker of TNF and other pro-Inflammatory biomarkers. Br J Pharmacol. 2013;169:1672–92. 23425071 10.1111/bph.12131 PMC3753829

[35] Abdel Ghafar HH, Ali GAM, Fouad OA, Makhlouf. SA enhancement of adsorption efficiency of methylene blue on Co_3_O_4_/SiO_2_ nanocomposite. Desalin Water Treat. 2013;53:2980–9.

[36] Lo S-F, Wang S-Y, Tsai M-J, Lin L-D. Adsorption capacity and removal efficiency of heavy metal ions by Moso and Ma bamboo activated carbons. Chem Eng Res Des. 2012;90:1397–406.

[37] El-Gamal SMA, Amin MS, Ahmed MA. Removal of Methyl orange and bromophenol blue dyes from aqueous solution using Sorel’s cement nanoparticles. J Environ Chem Eng. 2015;3:1702–12.

[38] Yagub MT, Sen TK, Afroze S, Ang HM. Dye and its removal from aqueous solution by adsorption: A review. Adv Colloid Interf Sci. 2014;209:172–84. 10.1016/j.cis.2014.04.002 24780401

[39] Özacar M, Şengil. IA adsorption of acid dyes from aqueous solutions by calcined alunite and granular activated carbon. Adsorption. 2002;8:301–8.

[40] Malik PK. Use of activated carbons prepared from sawdust and rice-husk for sorption of acid dyes: a case study of acid yellow 36. Dyes Pigm. 2003;56:239–49.

[41] Cheng J, Zhan C, Wu J, Cui Z, Si J, Wang Q, Peng X, Turng. LS highly efficient removal of methylene blue dye from an aqueous solution using cellulose acetate nanofibrous membranes modified by polydopamine. ACS Omega. 2020;5:5389–400. 32201829 10.1021/acsomega.9b04425 PMC7081408

[42] Mogharbel RT, Al-Hossainy AF, Qasim EA, Wahman AY, Farhan N, Ibrahim SM. Spectroscopic, kinetics and molecular structure investigations of acriflavine hydrochloride dye onto thiobarbituric acid nanoblend TD-DFT calculations: removal of dyes from wastewater applications. J Mol Liq. 2023;386:12249.

[43] El-Aal MA, Mogharbel RT, Ibrahim A, Almutlaq N, Zoromba, MSh, Al-Hossainy AF, Ibrahim SM. Synthesis, characterization, and photosensitizer applications for dye-based on ZrO_2_- acriflavine nanocomposite thin film [ZrO_2_ + ACF]^C^. J Mol Struct. 2022;1250:131827.

[44] Liang S, Guo X, Feng N, Tian Q. Isotherms, kinetics and thermodynamic studies of adsorption of Cu^2+^ from aqueous solutions by Mg^2+^/K^+^ type orange Peel adsorbent. J Hazard Mater. 2010;174:756–62. 19853995 10.1016/j.jhazmat.2009.09.116

[45] Ibrahim SM, Al-Hossainy AF. Synthesis, structural characterization, DFT, kinetics and mechanism of oxidation of bromothymol Blue: application to textile industrial wastewater treatment. Chem Pap. 2021;75:297–309.

[46] Al-Hossainy AF, Ibrahim A, Mogharbel RT, Ibrahim. SM synthesis of novel keto-bromothymol blue in different media using oxidation–reduction reactions: combined experimental and DFT-TDDFT computational studies. Chem Pap. 2021;75:3103–18.

[47] Akbaria A, Sabouria Z, Hosseini HA, Hashemzadeh A, Khatami M, Darroudi. M effect of nickel oxide nanoparticles as a photocatalyst in dyes degradation and evaluation of effective parameters in their removal from aqueous environments. Inorg Chem Commun. 2020;115:107867.

[48] Al-Aoh HA. Adsorption performances of nickel oxide nanoparticles (NiO NPs) towards bromophenol blue dye (BB). J Desalin Water Treat. 2018;110:229–38.

[49] Ho YS, McKay G, Wase DAJ, Foster. CF study of the sorption of divalent metal ions on to peat. Adsorpt Sci Technol. 2000;18:639–50.

[50] Bhattacharyya KG, Sharma. A kinetics and thermodynamics of methylene blue sorption on Neem (azadirachta indica) leaf powder. Dyes Pig. 2005;65:51–9.

[51] Ibrahim SM, Saad N, Ahmed MM, Abd El–Aal. M novel synthesis of antibacterial pyrone derivatives using kinetics and mechanism of oxidation of Azithromycin by alkaline permanganate. Bioorg Chem. 2022;119:105553. 34920338 10.1016/j.bioorg.2021.105553

[52] Hassanien R, Hassan ZA, Al-Assy W, Ibrahim. SM removal of toxic thymol sulfone phthalein dye from wastewater by using efficient adsorbent NiO nanoparticles. J Mol Struct. 2022;1269:133864.

[53] Kumar KV, Kumaran. A removal of methylene blue by Mango seed kernel powder. Biochem Eng J. 2005;27:83–93.

[54] Hassan RM, Ibrahim SM, Zaafarany IA, Fawzy A, Takagi. HD Base-catalyzed oxidation: kinetics and mechanism of hexacyanoferrate (III) oxidation of Methyl cellulose polysaccharide in alkaline solutions. J Mol Catal A: Chem. 2011;344:93–8.

[55] Hassan RM, Ibrahim. SM oxidation of some sustainable sulfated natural polymers: kinetics and mechanism of oxidation of water-soluble chondroitin-4-sulfate polysaccharide by hexachloroiridate (IV) in aqueous solutions. ACS Omega. 2019;4:2463–71. 31459485 10.1021/acsomega.8b02184 PMC6649260

[56] Hassan RM, Ibrahim SM. Base-catalyzed oxidation of sulfated kappa-carrageenan by alkaline hexacyanoferrate (III): A mechanistic approach of electron-transfer process. J Mol Liq. 2019;273:177–82.

[57] Hassan RM, Dahy AA, Ibrahim SM, Zaafarany IA, Fawzy. A oxidation of some macromolecules. Kinetics and mechanism of oxidation of Methyl cellulose polysaccharide by permanganate ion in acid perchlorate solutions. Ind Eng Chem Res. 2012;51:5424–32.

[58] Hassan RM, Takagi HD, Ibrahim SM. Orientation on the mechanistics of electron-transfer on oxidation of chondroitin-4-sulfate as sustainable sulfated polysaccharide by permanganate ion in aqueous perchlorate solutions. J Renew Mater. 2020;8:205–18.

[59] Hassan RM, Ibrahim SM, Sayed SA, Zaafarany IA. Promising biocompatible, biodegradable, and inert Polymers for purification of wastewater by simultaneous removal of carcinogenic cr (VI) and present toxic heavy metal cations: reduction of chromium (VI) by Poly (ethylene glycol) in aqueous perchlorate solutions. ACS Omega. 2020;5:4424–32. 32175490 10.1021/acsomega.9b03485 PMC7066562

[60] Hassan RM, Ibrahim SM, Takagi HD, Sayed. SA kinetics of corrosion Inhibition of aluminum in acidic media by water-soluble natural polymeric chondroitin-4-sulfate as anionic polyelectrolyte inhibitor. Carbohyd Poly. 2018;192:356–63. 10.1016/j.carbpol.2018.03.066 29691031

[61] Hassan RM, Ibrahim SM, Salman SA, Takagi. HD A promising water-soluble synthetic Polymer of high efficiency and low cost as inhibitor for Inhibition of metals corrosion: Inhibition of magnesium corrosion by Poly (ethylene glycol) in acidic media. J Bio- Tribo-Corrosion. 2019;5:101: 1–10.

[62] Ibrahim SM, Althagafi I, Takagi HD, Hassan RM. Kinetics and mechanism of oxidation of chondroitin-4-sulfate polysaccharide as a sulfated polysaccharide by hexacyanoferrate (III) in alkaline solutions with synthesis of novel coordination biopolymer chelating agent. J Mol Liq. 2017;244:353–9.

[63] Ibrahim SM, Al-Hossainy. AF kinetics and mechanism of oxidation of bromothymol blue by permanganate ion in acidic medium: application to textile industrial wastewater treatment. J Mol Liq. 2020;318:114041.

[64] Xiao J, Lv W, Xie Z, Tan Y, Song Y, Zheng. Q environmentally friendly reduced graphene oxide as a broad-spectrum adsorbent for anionic and cationic dyes via π–π interactions. J Mater Chem A. 2016;4:12126–35.

